# Cooperative Interaction between Phosphorylation Sites on PERIOD Maintains Circadian Period in *Drosophila*

**DOI:** 10.1371/journal.pgen.1003749

**Published:** 2013-09-26

**Authors:** David S. Garbe, Yanshan Fang, Xiangzhong Zheng, Mallory Sowcik, Rana Anjum, Steven P. Gygi, Amita Sehgal

**Affiliations:** 1 Department of Neuroscience, University of Pennsylvania School of Medicine, Translational Research Center, Philadelphia, Pennsylvania, United States of America; 2 Department of Cell Biology, Harvard Medical School, Boston, Massachusetts, United States of America; 3 Howard Hughes Medical Institute, University of Pennsylvania School of Medicine, Translational Research Center, Philadelphia, Pennsylvania, United States of America; Northwestern University, United States of America

## Abstract

Circadian rhythms in *Drosophila* rely on cyclic regulation of the *period* (*per*) and *timeless* (*tim*) clock genes. The molecular cycle requires rhythmic phosphorylation of PER and TIM proteins, which is mediated by several kinases and phosphatases such as Protein Phosphatase-2A (PP2A) and Protein Phosphatase-1 (PP1). Here, we used mass spectrometry to identify 35 “phospho-occupied” serine/threonine residues within PER, 24 of which are specifically regulated by PP1/PP2A. We found that cell culture assays were not good predictors of protein function in flies and so we generated *per* transgenes carrying phosphorylation site mutations and tested for rescue of the *per^01^* arrhythmic phenotype. Surprisingly, most transgenes restore wild type rhythms despite carrying mutations in several phosphorylation sites. One particular transgene, in which T610 and S613 are mutated to alanine, restores daily rhythmicity, but dramatically lengthens the period to ∼30 hrs. Interestingly, the single S613A mutation extends the period by 2–3 hours, while the single T610A mutation has a minimal effect, suggesting these phospho-residues cooperate to control period length. Conservation of S613 from flies to humans suggests that it possesses a critical clock function, and mutational analysis of residues surrounding T610/S613 implicates the entire region in determining circadian period. Biochemical and immunohistochemical data indicate defects in overall phosphorylation and altered timely degradation of PER carrying the double or single S613A mutation(s). The PER-T610A/S613A mutant also alters CLK phosphorylation and CLK-mediated output. Lastly, we show that a mutation at a previously identified site, S596, is largely epistatic to S613A, suggesting that S613 negatively regulates phosphorylation at S596. Together these data establish functional significance for a new domain of PER, demonstrate that cooperativity between phosphorylation sites maintains PER function, and support a model in which specific phosphorylated regions regulate others to control circadian period.

## Author Summary

Circadian rhythms coordinate an organism's activities with its environment to ensure appropriate physiology and behavior at the relevant times of day. In *Drosophila melanogaster*, the central molecular clock is regulated by transcriptional and translational feedback loops that drive rhythmic waves of gene expression. Additionally, the clock is controlled by post-translational modifications, including phosphorylation of a core negative regulator, PERIOD (PER). Using a proteomic approach, we identified two key phosphorylation sites within PER that cooperate to establish proper daily rhythms. Mutation of both residues results in a period of ∼30 hrs, whereas single mutant phenotypes are less severe suggesting these sites normally cooperate to control the pace of the clock. Biochemical and immunohistochemical data demonstrate that altered PER phosphorylation and changes in protein stability give rise to behavioral defects. Other mutations in the vicinity of the two sites above also alter circadian period, thereby confirming a new regulatory domain of PER. Our results also suggest that the phospho-residues identified in this study regulate phosphorylation in another important domain of PER. Together our data establish that cooperativity between neighboring phospho-sites as well as interactions between different phosphorylated domains of PER control its stability and overall circadian periodicity.

## Introduction

Circadian rhythms, exhibited by a variety of organisms ranging from bacteria to plants and humans, help to establish daily temporal organization. These rhythms are typically generated by one or more feedback loops within core clock or “pacemaker” cells. In *Drosophila* the major loop consists of the *period* (*per*) and *timeless* (*tim*) genes, which autoregulate their expression by inhibiting activity of their transcriptional activators, the CLOCK (CLK) and CYCLE (CYC) proteins at distinct times of day, thereby driving their own rhythmic expression as well as that of other clock-controlled genes [1], [2]. Studies in *Drosophila* have been instrumental in not only identifying components of core clock machinery, but also for teasing apart more intricate regulatory mechanisms. Additionally, discovery of core clock genes in *Drosophila* ultimately led to identification of their human orthologs and the discovery that some of these genes are mutated in human circadian disorders [3].

Levels of PER and TIM cycle over the course of the day with peak expression occurring in the mid- to late-night. TIM levels decline at the end of the night, and decrease rapidly following daybreak due to light-dependent degradation [4], followed by a slower decrease in PER levels. Degradation of PER and TIM allows transcription to start anew, but their protein levels do not accumulate immediately. As PER expression begins to increase during the middle-late day, it is phosphorylated by DOUBLETIME (DBT) and turned over. It is only after TIM accumulates and heterodimerizes with PER at the end of the day that PER is protected from degradation. Stable nuclear expression of TIM and PER later in the night leads to a decrease in CLK/CYC heterodimer activity [1].

It is clear that cyclic phosphorylation of core clock proteins is critical for generating a functional clock. For example, temporal changes in the state of PER phosphorylation regulate various aspects of PER function including protein stability, subcellular localization, protein-protein interactions, and transcriptional repression [2], [5], [6]. Moreover, kinases, such as DOUBLETIME [(DBT); homolog of mammalian casein kinase I (CK1)], CK2, SHAGGY [(SGG); homolog of Glycogen Synthase Kinase-3β (GSK-3β)], and NEMO/NLK, and protein phosphatases such as Protein Phosphatase-2A (PP2A) and Protein Phosphatase-1 (PP1), regulate PER phosphorylation/dephosphorylation and overall time-keeping mechanisms [7]–[14]. While the physiological consequences of PER phosphorylation and dephosphorylation are starting to be elucidated, mechanistic details remain poorly understood.

In this study, using a proteomic approach to identify phospho-occupied sites, we demonstrate that phospho-residues PER-T610, and to a larger extent PER-S613, act as key regulators of the *Drosophila* clockwork and help to control timing of phosphorylation, overall PER stability, and circadian periodicity. Importantly, S613 is conserved from flies to mammals and thus may have implications for human circadian disorders. Mutating T610 and S613 to alanine leads to a long daily period length due to a delay in PER protein degradation and changes in CLK phosphorylation and CLK-mediated output. Notably, it appears as though these two residues act cooperatively to modulate PER function. Additionally, our data suggest S613 impacts clock speed by regulating the well-known Per-Short domain.

## Results

### Mass spectrometry identifies 35 Serine/Threonine phospho-residues in *Drosophila* PER

**Figure 1:**
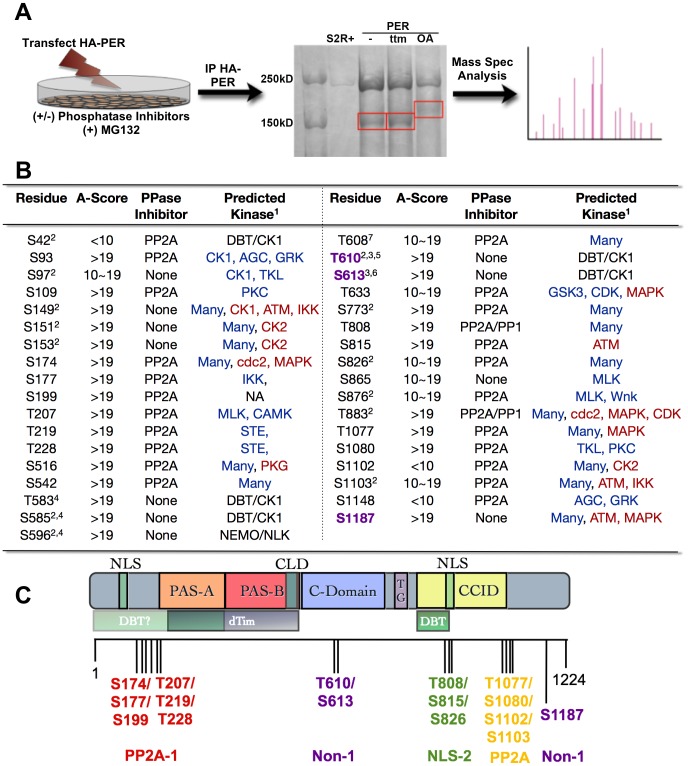
PER phosphorylation sites mapped by mass spectrometry. (**A**) Diagram depicting mass spectrometry experimental approach. See Material and Methods for additional details. (**B**) Table of phosphorylated residues of PER identified in this study. The exact phosphorylation sites are shown as serine (S) or threonine (T) followed by the number of their position in the PER protein sequence (1–1224, Accession #P07663). AScore was determined as described in Beausoleil,S.A. 2006 [29]. In general, phosphorylation sites detected by mass spec with an AScore>19 are almost certain; with 10<AScore<19 are probable; with AScore<10, are detected but uncertain. PPase inhibitor indicates whether a residue is naturally phosphorylated in S2R+ cells (None), or is phosphorylated only when treated with PP1 and/or PP2A inhibitors. 1 = The GPS (blue text; high threshold) and KinasePhos (red text; 100% specificity) online databases were used to predict kinase activity except where noted; 2 = Chiu et al., 2008; 3 = Kivimae et al., 2008; 4 = Chiu et al., 2011; 5 = Additional predictions: KinasePhos = cdc2, MAPK, CDK. Many other kinases were predicted to phosphorylate T610 by GPS; 6 = Additional predictions: GPS = AGC; KinasePhos = MAPK at 90% specificity; 7 = Note, S607 was identified to be a DBT substrate in Kivimae et al., 2008. Abbreviations: MLK = Mixed Lineage Kinase; TKL = Toll Kinase-Like; GRK = GPCR Kinase; AGC = PKA/G/C Kinase Group (**C**) Schematic representation of PER including functional domains and grouped phosphorylation sites (adapted from [32], with updates from [26], [33]). The nuclear localization signal (NLS), two PER-ARNT-SIM (PAS) domains (PAS-A and PAS-B), the cellular localization domain (CLD), the conserved C-domain, the threonine-glycine (TG) rich region, and dCLK:CYC inhibition domain (CCID) are shown. The shaded boxes below PER indicate regions through which it interacts with other clock proteins: DBT = DOUBLETIME and TIM = TIMELESS. Vertical black lines indicate approximate positions of phosphorylated residues. Red, purple, green, and yellow groups of residues correspond to the transgenic lines used in Figure 3. PP2A-1/2 = Residues phosphorylated only in the presence of the PP2A inhibitor; NLS = Residues located near Nuclear Localization Signal; Non-1 = Naturally phosphorylated residues in presence or absence of phosphatase inhibitors.

**Figure 2:**
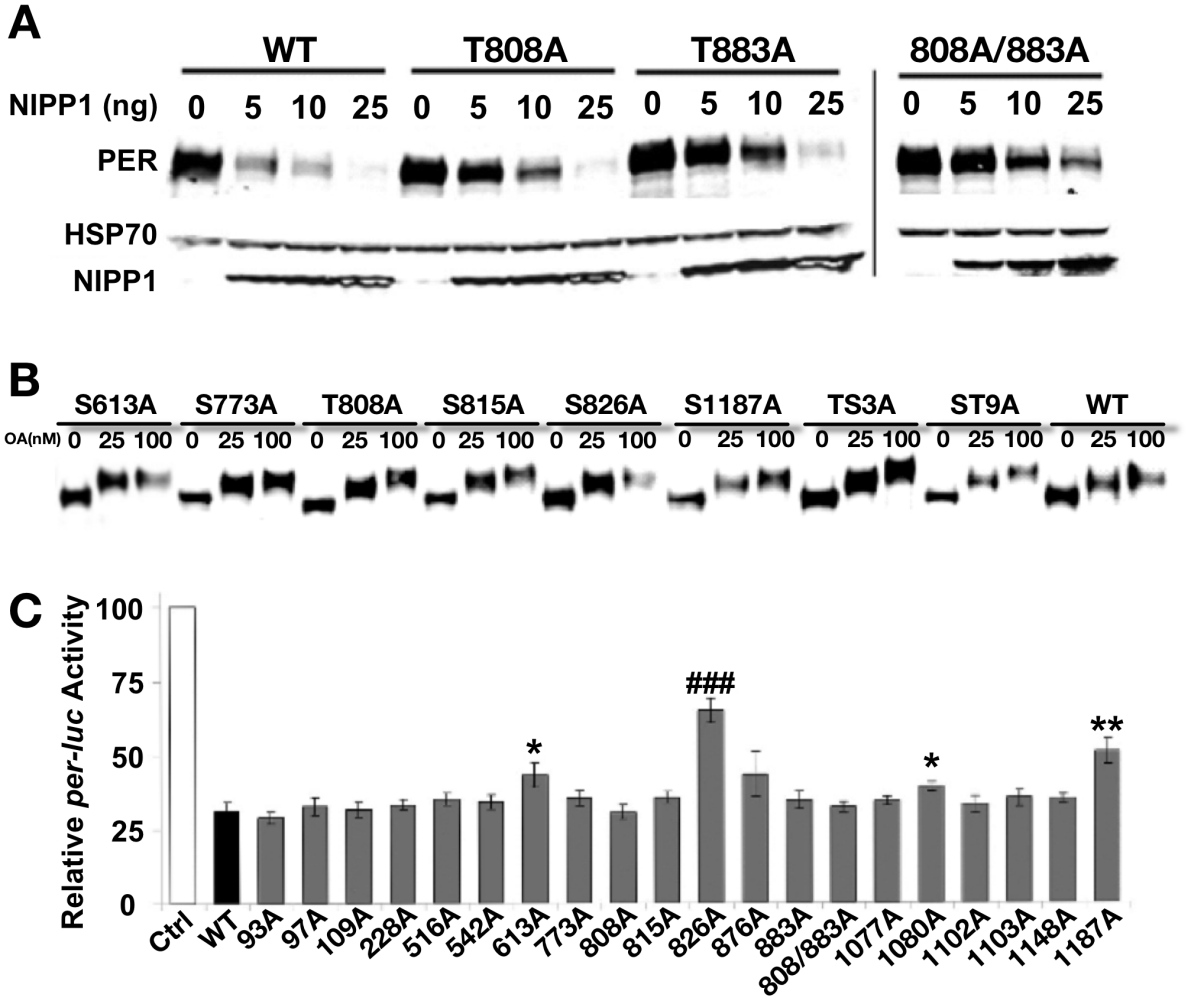
Analysis of PER phosphorylation sites in S2 cells. (**A**) Mutating PP1 target sites increases PER stability. Mass spec screen detected two phosphorylated Threonine (T) residues, T808 and T883, when PP1 was inhibited in the S2R+ cells. They were mutated to nonphosphorylatable alanine (A) individually or together in the full-length PER (T808A, T883A and T808A/883A). The response to inhibition of PP1 (by co-transfecting NIPP1) was determined for mutant and wild-type PER (WT). The HSP70 band is shown as a loading control. Inhibition of PP1 induces PER degradation in both WT and mutant PERs, but mutants show a relatively higher tolerance to PP1 inhibition and more so with the T808A/883A double mutant. (**B**) Inhibition of PP2A by increased dosage of Okadaic Acid (OA) induces robust mobility shifts in both WT and all mutant PERs tested, including ones containing up to nine mutated phosphorylation sites. We tested the following individual mutations in this assay and observed the same result: S93A, S97A, S109A, T228A, S516A, S542A, S613A, S773A, T808A, T815A, S826A, S876, T883A, T1077A, S1080A, S1102A, S1103A, S1148A, and S1187A (some data not shown). PER-TS3A includes T763A/T765A/S767A [33] and PER-ST9A includes S756A/T763A/T765A/S767A/S787A/T791A/S795A/S798A/T802A [26]. Proteasome inhibitor MG132 (100 µM) was added to protect phosphorylated PER from degradation. The results shown are representative of two independent experiments. (**C**) Repression activity of WT and mutant PERs in an S2R+ cell based *per*-*luciferase* (*per-luc*) reporter assay. The graph shows average percent luciferase activity ± s.e.m, which is normalized to the control cells that were transfected with pAc-*clk*-HA and *per-luc* but not pAc-*per*-HA. This is inversely related to the repression activity of each PER (WT and mutants) transfected. Results are pooled from seven independent experiments and each PER construct was examined independently at least four times. Statistical significance is determined by one-tailed t-test with unequal variance at: P*<0.01, P**<0.001, and P^###^<0.000001. Of the mutant dPERs tested, PER-S613A, -T826A, -S1080A and –S1187A display a significant decrease in repressor activity, with PER-T826A exhibiting the most robust change and significance.

Despite efforts to better understand the role of cyclic phosphorylation in regulating the circadian clock, the complete repertoire of phosphorylation residues within PER has not been identified. Additionally, how phosphorylation contributes to overall circadian regulation is still being investigated. While some phospho-sites have been determined [11], [15]–[17] nothing is known about phosphatase targets. To map phosphorylation sites of PER, in particular those modified by protein phosphatases, we expressed an HA-tagged PER in S2 cells in the presence or absence of phosphatase inhibitors, immunoprecipitated PER-HA, and conducted mass spectrometry analysis (Figure 1A). In total, we identified 35 Serine (S) and Threonine (T) residues spanning PER that were phosphorylated under our conditions (Figure 1B). Twenty-four residues (S42, S93, S109, S174, S177, S199, T207, T219, T228, S516, S542, T608, T633, S773, T808, S815, S826, S876, T883, T1077, S1080, S1102, S1103, S1148) were specifically phosphorylated in the presence of the PP2A inhibitor, Okadaic Acid (OA) suggesting that PP2A controls the phospho-occupancy of these sites (Figures 1B and 2B). Two of the PP2A sites, T808 and T883, were also phosphorylated in the presence of the PP1-selective inhibitors, Tautomycin or NIPP1 (Figures 1B and 2A). Eleven residues (S97, S149, S151, S153, T583, S585, S596, T610, S613, S865, and S1187) were phosphorylated in the presence or absence of both phosphatase inhibitors, suggesting they are relatively resistant to the action of either phosphatase. For simplicity, we hereafter call these three types of residues “PP2A-”, “PP1-”, or “Non-” sites respectively (Figure 1C).

### Deciphering phospho-residues critical for PER function

We followed up the mass spectrometry analysis by mutating individual phospho-sites and testing them in cell culture assays. We first asked if any of the PP2A- or PP1-regulated sites are important for overall PER stability in cells. As shown in Figure 2A and consistent with our previous data [13], increasing PP1 inhibition (increasing concentrations of NIPP1) leads to an increase in PER degradation, supporting the idea that PP1 activity (i.e. removal of specific phosphate modifications) is critical for maintaining PER levels in the cell [13]. Only two sites were identified as PP1 targets, which is consistent with the lack of a mobility shift in PER upon PP1 inhibition (Figure 2A and [13]). Mutating either or both PP1-regulated sites identified in this study (T808 and T883) to alanine increases overall PER stability in the presence of NIPP1, supporting the idea that phosphorylation at these two sites decreases PER stability, and is typically targeted by PP1 (Figure 2A). Interestingly, while inhibition of PP2A leads to a decrease in PER mobility through the gel, this inhibition does not generally affect PER stability, although mutations in a few specific PP2A-targeted sites may have minor effects (Figure 2B and [13]). Additionally, we note that mutation of individual PP2A-regulated residues does not impact the overall phosphorylation state of PER as judged by PER mobility through the gel (Figure 2B). Thus, mobility shifts may be produced by multiple PP2A-targeted sites, or by one not identified in this study. Higher concentrations of the PP2A inhibitor, Okadaic acid, produce larger shifts, perhaps due to inhibition of other phosphatases.

**Figure 3:**
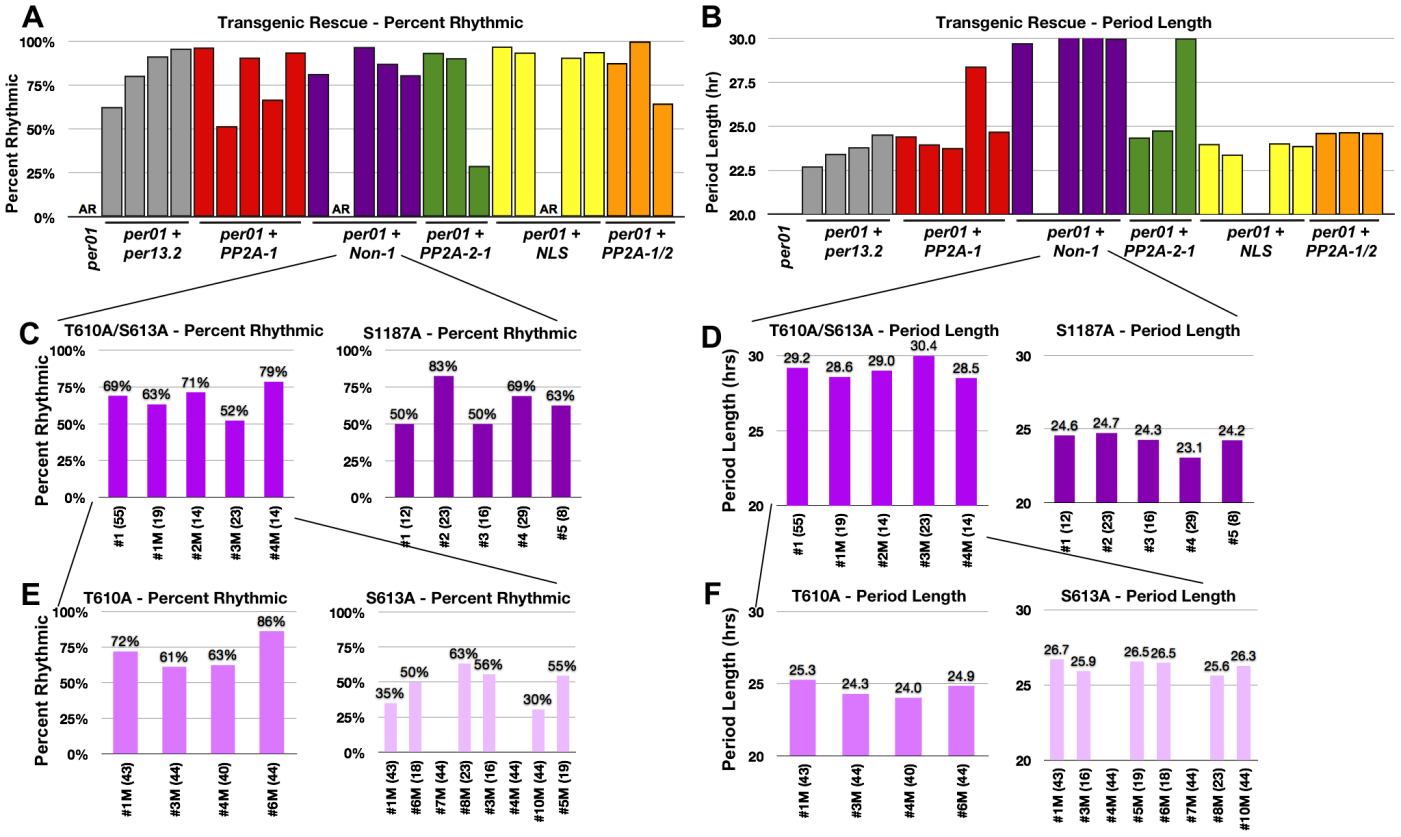
Residues T610 and S613 cooperate to control period length. (**A and B**). Graphs depicting percent rhythmic (A) and period length (B) of various transgenic lines containing groups of phospho-mutations highlighted in Figure 1. Non-1 transgenic flies, containing only three mutant residues, exhibit a period length to ∼29–30 hours (purple bars in B). The long period phenotype exhibited by one individual line from PP2A-1 and PP2A-2 (panel B, Table 1) are probably not meaningful since rhythmicity of these lines is low and additional transgenic lines of the same construct exhibit normal PER function. While not tested, perhaps PER protein levels in these lines are lower than the others. (**C and D**) Graphs depicting percent rhythmic (C) and period length (D) of transgenic lines harboring either the T610A/S613A mutations or the single S1187A mutation. The T610A/S613A double mutant transgene retains the long period phenotype (D). (**E and F**) Graphs depicting percent rhythmic (E) and period length (F) of transgenic lines harboring either the single T610A or single S613A mutation. Note, neither single mutant displays a period length as long as the double mutant suggesting they function cooperatively to maintain wild type period length. In all graphs, period lengths are listed above each bar and values in parentheses indicate the number of flies analyzed. Variation in strength of rescue is attributed to differences in genomic insertion sites; data from all lines are shown. Raw data is summarized in Table 1. Only male flies were analyzed in these experiments.

We also tested individual phospho-site mutant constructs for PER repressor function in a *per-luciferase* (*per-luc*) cell culture assay. In this assay, *per-luc* activity is induced by CLK, and the CLK activity can be inhibited by PER. We found that individually mutating S613, S826, S1080, or S1187 to alanine leads to significantly reduced repression in the *per-luc* assay (Figure 2C). PER-S826A shows the most robust and consistent effect on CLK-mediated repression and a serine-aspartic acid change at this site also increases nuclear localization of PER, suggesting that phosphorylation at this site promotes nuclear entry (Figure 2C and data not shown). However, transgenic rescue of *per^01^* flies indicates that the S826A mutant protein retains wild type PER function and drives normal daily activity rhythms *in vivo* [transgenic rescue of lines containing the S826A mutation in combination with other mutant residues is shown in Figure 3A and B (NLS-1), while rescue data for S826A alone is shown in Table 1)]. Similarly, transgenes containing the S1080A and S1187A mutations also largely retain PER function, with S1187A producing at best a slightly longer period in *per^01^* mutants (Figure 3C–D and data not shown). Note, these data do not exclude the possibility that these sites are important for other aspects of PER function, for example in peripheral clocks. We note also that effects of various mutations on the repressor function of PER in the *per-luc* assay could result from small changes in stability in S2 cells (Figure 2). Finally, we cannot rule out the possibility that changes in the *per-luc* assay reflect true, but small changes in CLK-mediated transcription; however, these are likely not physiologically relevant as we do not see behavioral defects in transgenic flies. The final mutation that significantly, although slightly, reduces PER's ability to repress *per-luc* activity in cells, S613A, was also identified through a different strategy described below.

**Table 1:** Locomotor activity rhythms of *per^01^* mutants and transgenic lines used in this study (see Figures 3, 7 and 8).

Genotype	% Rhythmic	Period, hr (± SD)	FFT (± SD)	n =
*per^01^*	AR	NA	NA	58
*per^01^*+*per-13.2A*	62.5	22.73 (0.22)	0.058 (0.028)	16
*per^01^*+*per-13.2B*	80.3	23.44 (0.28)	0.059 (0.034)	76
*per^01^*+*per-13.2C*	91.3	23.82 (0.25)	0.078 (0.049)	23
*per^01^*+*per-13.2D*	95.7	24.54 (0.23)	0.082 (0.042)	23
*per^01^*+*per-PP2A-1 (#A)*	96.3	24.40 (0.23)	0.094 (0.043)	27
*per^01^*+*per-PP2A-1 (#B)*	51.6	23.98 (0.30)	0.068 (0.024)	31
*per^01^*+*per-PP2A-1 (#C)*	90.6	23.77 (0.24)	0.060 (0.032)	32
*per^01^*+*per-PP2A-1 (#D)* *	66.7	28.40 (1.44)	0.024 (0.024)	12
*per^01^*+*per-PP2A-1 (#F)*	93.5	24.70 (0.22)	0.070 (0.033)	31
*per^01^*+*per-Non-1 (#A)*	81.3	29.71 (0.44)	0.059 (0.035)	32
*per^01^*+*per-Non-1 (#B)*	AR	NA	NA	28
*per^01^*+*per-Non-1 (#C)*	96.6	30.33 (0.54)	0.092 (0.050)	29
*per^01^*+*per-Non-1 (#D)*	87.1	30.12 (0.61)	0.098 (0.097)	31
*per^01^*+*per-Non-1 (#E)*	80.6	29.99 (0.68)	0.079 (0.038)	31
*per^01^*+*per-PP2A-2 (#B)*	93.3	24.37 (0.21)	0.079 (0.052)	30
*per^01^*+*per-PP2A-2 (#1)*	90.3	24.77 (0.30)	0.057 (0.024)	31
*per^01^*+*per-PP2A-2 (#2)* *	29.0	30.01 (1.85)	0.039 (0.021)	31
*per^01^*+*per-NLS (#A)*	96.6	24.0 (0.18)	0.083 (0.033)	29
*per^01^*+*per-NLS (#B)*	93.5	23.4 (0.25)	0.059 (0.029)	31
*per^01^*+*per-NLS (#C)*	AR	NA	NA	22
*per^01^*+*per-NLS (#D)*	90.6	24.04 (0.24)	0.078 (0.041)	32
*per^01^*+*per-NLS (#E)*	93.8	23.91 (0.19)	0.089 (0.033)	24
*per^01^*+*per-PP2A-1/2 (#A)*	87.5	24.63 (0.24)	0.058 (0.026)	24
*per^01^*+*per-PP2A-1/2 (#1)*	100	24.67 (0.22)	0.075 (0.036)	32
*per^01^*+*per-PP2A-1/2 (#2)*	64.5	24.63 (0.35)	0.041 (0.022)	31
*per^01^*+*per-T610A/S613A(#1)*	69.1	29.19 (1.38)	0.035 (0.014)	55
*per^01^*+*per-T610A/S613A(#1M)*	63.2	28.58 (0.45)	0.037 (0.014)	19
*per^01^*+*per-T610A/S613A(#2M)*	71.4	29.00 (0.38)	0.035 (0.026)	14
*per^01^*+*per-T610A/S613A(#3M)*	52.2	30.39 (0.53)	0.031 (0.008)	23
*per^01^*+*per-T610A/S613A(#4M)*	78.6	28.51 (0.52)	0.039 (0.016)	14
*per^01^*+*per-S613A (#1M)*	34.9	26.7 (0.38)	0.030 (0.013)	43
*per^01^*+*per-S613A (#3M)*	50.0	25.92 (0.31)	0.033 (0.008)	16
*per^01^*+*per-S613A (#4M)*	AR	NA	NA	44
*per^01^*+*per-S613A (#5M)*	63.2	26.55 (0.19)	0.028 (0.008)	19
*per^01^*+*per-S613A (#6M)*	55.6	26.47 (0.26)	0.041 (0.016)	18
*per^01^*+*per-S613A (#7M)*	AR	NA	NA	44
*per^01^*+*per-S613A (#8M)*	30.4	25.62 (0.13)	0.028 (0.007)	23
*per^01^*+*per-S613A (#10M)*	54.5	26.26 (0.34)	0.034 (0.013)	44
*per^01^*+*per-T610A (#1M)*	72.1	25.27 (0.33)	0.068 (0.032)	43
*per^01^*+*per-T610A (#3M)*	61.4	24.29 (0.35)	0.034 (0.010)	44
*per^01^*+*per-T610A (#4M)*	62.5	24.03 (0.29)	0.036 (0.015)	40
*per^01^*+*per-T610A (#6M)*	86.4	24.85 (0.27)	0.055 (0.023)	44
*per^01^*+*per-S1187A(#1M)*	50.0	24.57 (0.50)	0.059 (0.023)	12
*per^01^*+*per-S1187A(#2M)*	82.6	24.73 (0.53)	0.041 (0.012)	23
*per^01^*+*per-S1187A(#3M)*	50.0	24.26 (0.44)	0.048 (0.019)	16
*per^01^*+*per-S1187A(#4M)*	69.0	24.06 (0.35)	0.040 (0.011)	29
*per^01^*+*per-S1187A(#5M)*	62.5	24.23 (0.33)	0.052 (0.013)	8
*per^01^*+*per-P611A(#1M)*	100	26.72 (0.45)	0.065 (0.026)	22
*per^01^*+*per-P611A(#3M)*	95.8	26.91 (0.27)	0.068 (0.036)	24
*per^01^*+*per-P611A(#4F)*	100	26.77 (0.25)	0.078 (0.035)	20
*per^01^*+*per-P612A(#2M)*	91.7	26.81 (0.34)	0.049 (0.021)	24
*per^01^*+*per-P612A(#4M)*	90	27.54 (0.28)	0.080 (0.056)	10
*per^01^*+*per-P612A(#5M)*	95.8	26.93 (0.26)	0.100 (0.038)	24
*per^01^*+*per-P612A(#6M)*	100	27.35 (0.23)	0.086 (0.041)	18
*per^01^*+*per-P612A(#7F)*	100	26.87 (0.34)	0.070 (0.040)	22
*per^01^*+*per-Y614A(#1M)*	94.4	25.52 (0.25)	0.044 (0.016)	18
*per^01^*+*per-Y614A(#2M)*	66.7	25.41 (0.39)	0.049 (0.025)	24
*per^01^*+*per-Y614A(#3M)*	73.9	25.33 (0.39)	0.044 (0.022)	23
*per^01^*+*per-S826A*	73.7	23.6 (0.26)	0.040 (0.027)	38
*per^01^*+*per-S596A (#1M)*	100	15.7 (0.24)	0.103 (0.044)	17
*per^01^*+*per-S596A (#2M)*	83.3	17.3 (0.49)	0.061 (0.040)	24
*per^01^*+*per-S596A (#5M)*	87.5	16.7 (0.39)	0.047 (0.026)	24
*per^01^*+*per-S596A (#6M)*	AR	NA	NA	24
*per^01^*+*per-S596A+S613A (#1M)*	80.0	18.3 (0.44)	0.051 (0.031)	25
*per^01^*+*per-S596A+S613A (#2M)*	96.2	18.1 (0.28)	0.087 (0.030)	26
*per^01^*+*per-S596A+S613A (#3M)*	86.4	17.7 (0.24)	0.113 (0.042)	22
*per^01^*+*per-S596A+S613A (#4M)*	92.0	18.0 (0.23)	0.076 (0.040)	25
*per^01^*+*per-S596A+S613A (#5M)*	88.0	17.9 (0.17)	0.056 (0.035)	25
*per^01^*+*per-S596A+S613A (#6M)*	92.0	17.9 (0.38)	0.046 (0.023)	25
*per^01^*+*per-S596A+S613A (#7M)*	58.3	16.6 (0.14)	0.037 (0.026)	24
*per^01^*+*per-S596A+T610A/S613A (#1M)*	86.7	19.0 (0.36)	0.067 (0.029)	15
*per^01^*+*per-S596A+T610A/S613A (#2M)*	100	19.1 (0.31)	0.061 (0.035)	15
*per^01^*+*per-S596A+T610A/S613A (#4M)*	73.3	19.2 (0.26)	0.052 (0.024)	15
*per^01^*+*per-S596A+T610A/S613A (#5F)*	75.0	18.6 (0.14)	0.044 (0.022)	16
*per^01^*+*per-S589A (#1M)*	100	19.9 (0.21)	0.067 (0.021)	23
*per^01^*+*per-S589A (#2M)*	91.3	20.0 (0.20)	0.067 (0.043)	23
*per^01^*+*per-S589A (#3M)*	54.2	19.1 (0.22)	0.026 (0.019)	24
*per^01^*+*per-S589A (#4M)*	93.3	19.7 (0.20)	0.060 (0.023)	15
*per^01^*+*per-S589A (#5M)*	AR	NA	NA	17
*per^01^*+*per-S589A+S613A (#1M)*	76.0	22.3 (0.41)	0.050 (0.036)	25
*per^01^*+*per-S589A+S613A (#2M)*	24.0	22.6 (0.44)	0.024 (0.022)	25
*per^01^*+*per-S589A+S613A (#3F)*	AR	NA	NA	25
*per^01^*+*per-S589A+S613A (#5F)*	92.0	21.8 (0.41)	0.060 (0.033)	25
*per^01^*+*per-S589A+S613A (#6F)*	84.0	21.4 (0.19)	0.045 (0.032)	25
*per^01^*+*per-S589A+T610A/S613A (#3M)*	85.7	22.9 (0.32)	0.054 (0.021)	14
*per^01^*+*per-S589A+T610A/S613A (#4M)*	87.5	23.2 (0.32)	0.066 (0.049)	16
*per^01^*+*per-S589A+T610A/S613A (#6M)*	87.5	23.3 (0.32)	0.040 (0.015)	16
*per^01^*+*per-S589A+T610A/S613A (#7M)*	AR	NA	NA	16

AR = Arrhythmic (FFT<0.020); NA = Not Applicable due to arrhythmicity.

* Long period phenotype of lines PP2A-1#D and PP2A-2#2 is probably not meaningful since rhythmicity of these lines is low and additional transgenic lines of the same constructs exhibit normal PER function.

Together, the data above demonstrate that results from the commonly used *per-luc* cell culture repression assay do not always correlate with functional significance *in vivo*. Therefore, we used a different approach to identify relevant sites *in vivo*. We designated four distinct groups of residues and mutated multiple sites within each group (Figure 1C). Groups of residues were designated based on their proximity to each other as well as possible functional significance. For example, we identified two clusters of PP2A regulated sites, PP2A-1 and PP2A-2, located near the N- and C-terminus of the protein respectively, a group of residues near the Nuclear Localization Sequence (NLS), and residues that were unaffected by either PP2A or PP1 inhibition (Non-1). Using this approach we discovered that mutating residues within the PP2A-1, PP2A-2, or NLS-1 groups did not affect the ability of PER to rescue the *per^01^* null phenotype (Figure 3A and B). Significantly, it should be noted that a PER transgene containing up to 10 phospho-residue mutations in the same construct (PP2A1/2, orange bars, Figure 3A and B), is still able to rescue both arrhythmicity and period length of *per^01^* flies. These data, combined with the *in vitro* data described above (Figure 2), suggest that none of these specific PP2A-regulated sites are critical for normal PER function in regulating the central molecular clock within pacemaker neurons, although effects in other tissues (or under other conditions) cannot be excluded. On the other hand, we found that mutating three S/T residues to alanine within the Non-1 group (T610A, S613A, and S1187A; see Figure 1C) leads to a dramatic increase in period length (∼30 hour) in the *per^01^* background (Figure 3B, purple bars). These data suggest that phosphorylation at T610, S613, and/or S1187 normally speeds up the clock in *Drosophila*. As mentioned previously, S613 and S1187 were also identified in the *in vitro per-luc* assay. We note that some of the transgenic lines do not rescue activity rhythms, and the degree to which arrhythmicity is rescued differs between the transgenic lines studied. As these variations were also seen when testing wild type transgenes (Figure 1 and Table 1), we attribute them to differences in transgene insertion site within the genome and are showing data for all transgenic lines. Raw data from all lines are shown in Table 1.

### Blocking phosphorylation at T610 and S613 increases period length *in vivo*

In order to determine if any one of the three sites, T610, S613, or S1187, was individually responsible for the long period phenotype observed in the transgenic flies, we first generated flies containing the S1187A mutation separated from the T610A/S613A mutations. As shown in Figure 3D, individually mutating S1187 rescues arrhythmicity and does not have a large affect on period length suggesting this residue is not critical for PER-dependent timekeeping. Once again, these results reinforce the idea that data from the *in vitro per-luc* repression assay are not always consistent with physiological significance *in vivo*. On the other hand, flies harboring a T610A/S613A double mutant transgene retain the long (∼29–30 hour) period length (Figure 3D). It is unlikely that the long period phenotype observed in PER-T610A/S613A flies is a consequence of altered protein expression given that overall levels of wild type and PER-T610A/S613A proteins appear similar (see below) and that this defect is observed across multiple independent transgenic lines.

We then generated T610A or S613A single mutant transgenic flies to investigate the contribution from each individual phospho-residue. As expected, both single mutant transgenes rescue arrhythmicity (Figure 3E and Table 1) and flies containing the single T610A mutation have a near wild type period length (Figure 3F and Table 1). Interestingly, flies carrying the S613A mutant transgene have a period length of approximately 26–27 hours, a significantly long period, but shorter than the 29–30 hour period length seen in the double T610A/S613A mutant (Figure 3F and Table 1). These data demonstrate that T610 *and* S613 cooperate to control the overall speed of the clock and suggest that both residues function together to maintain daily rhythms, with S613 playing a larger role.

### Amino acid conservation in the T610/S613 region

**Figure 4:**
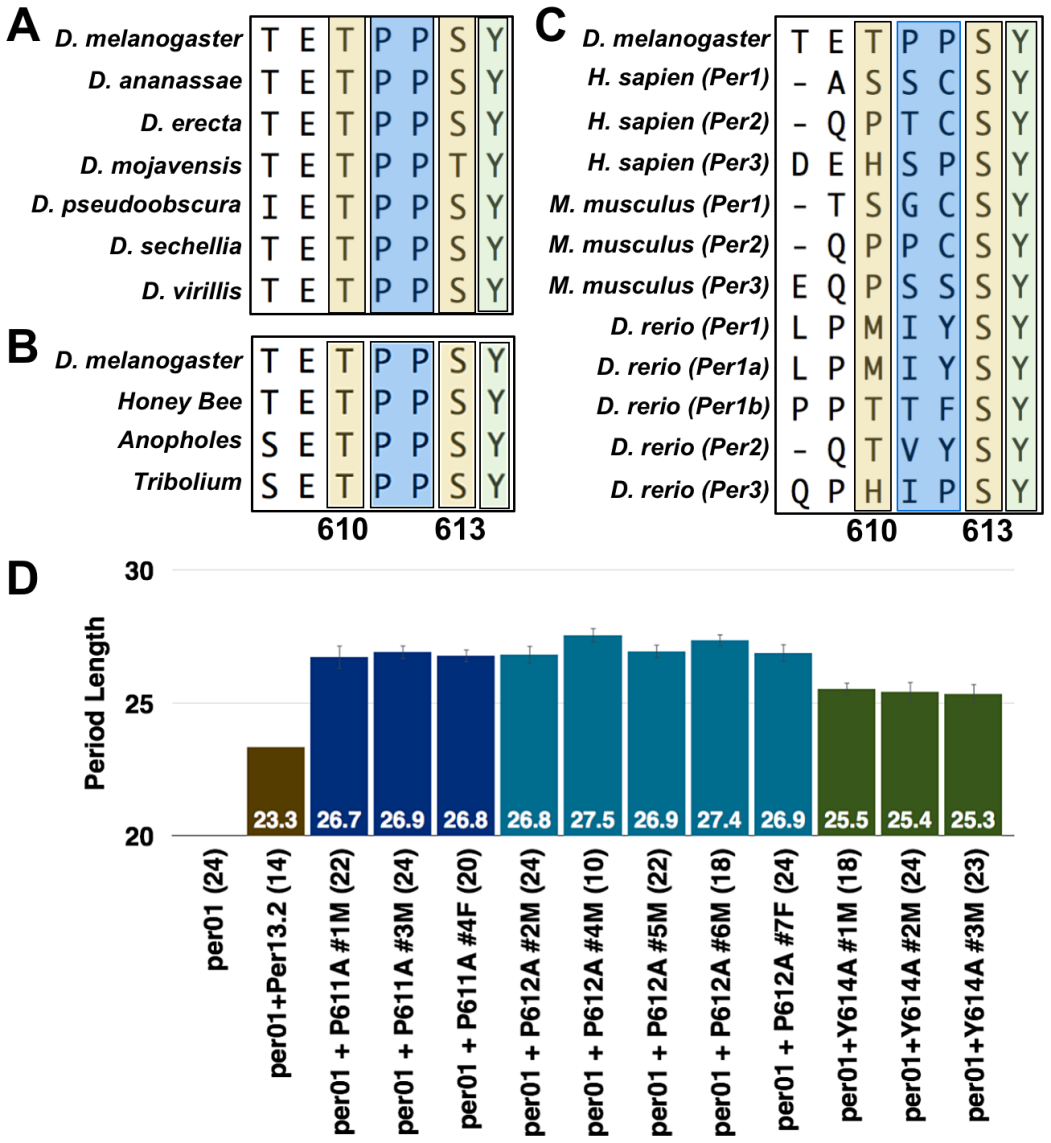
S613 is conserved throughout evolution. PER protein sequence alignment with various (**A**) *Drosophila*, (**B**) insect, and (**C**) vertebrate species. Alignments were generated using the Clustal-W Method within the MegAlign program contained in the DNA Star software package. Alignment Reports were exported and cropped to highlight the indicated residue stretches. Note the evolutionary conservation of the S613 (second yellow box) and Y614 (green box) residues; prolines are highlighted in a blue box. (**D**) Graphs depicting period length of a wild type transgenic fly line (brown bar) or those harboring the P611A (dark blue bars), P612A (light blue bars), or Y614A (green bars) mutations. Data from all transgenic lines are shown. Period lengths are listed within each bar and values in parentheses indicate the number of flies analyzed. Raw data are summarized in Table 1. Only male flies were analyzed in these experiments.

Having identified T610 and S613 as phospho-residues critical for normal PER function, we were curious to know if either site was conserved in other *Drosophila* or throughout evolution. Therefore, we aligned the PER protein sequence across a number of *Drosophila*, insect, and vertebrate species and discovered that S613 is conserved all the way to mammals (Figure 4A–C). This high degree of conservation suggests that S613 plays a critical role in PER function. As noted in Figure 4, T610 is also conserved across *Drosophila* and insect species but not in higher vertebrates.

During the sequence analysis, we noticed that tyrosine-614, immediately adjacent to S613, is also conserved throughout evolution (Figure 4A–C). As with the S613A mutation (Figure 3F), individually mutating Y614 to alanine rescues rhythmicity of *per^01^* mutants, but produces a long period phenotype (∼25.5 hrs; Figure 4B). These data demonstrate that Y614 is also likely an important residue regulating PER function throughout evolution.

Lastly, we observed that the two proline residues between T610 and S613 (P611/P612) are conserved in insects (Figure 4A and B). Previously, it was reported that phosphorylation of PER at S661 is regulated by a proline-directed kinase [14], so we were interested in determining if prolines 611 and 612 are important for T610 or S613 phosphorylation. Therefore, we individually mutated each proline and tested whether these mutant transgenes were able to rescue the *per^01^* mutant phenotype. Consistent with proline-directed phosphorylation of S613, transgenes containing individual P611A and P612A mutations are able to rescue rhythmicity of the *per* null phenotype, but give rise to a period length similar to the individual S613A mutations (∼27 hrs; Figure 4B). Together, our data demonstrate conservation of the region surrounding and containing T610 and S613 and support previous reports [17] that this is a key region governing proper PER regulation and function.

### S613A affects global PER phosphorylation

**Figure 5:**
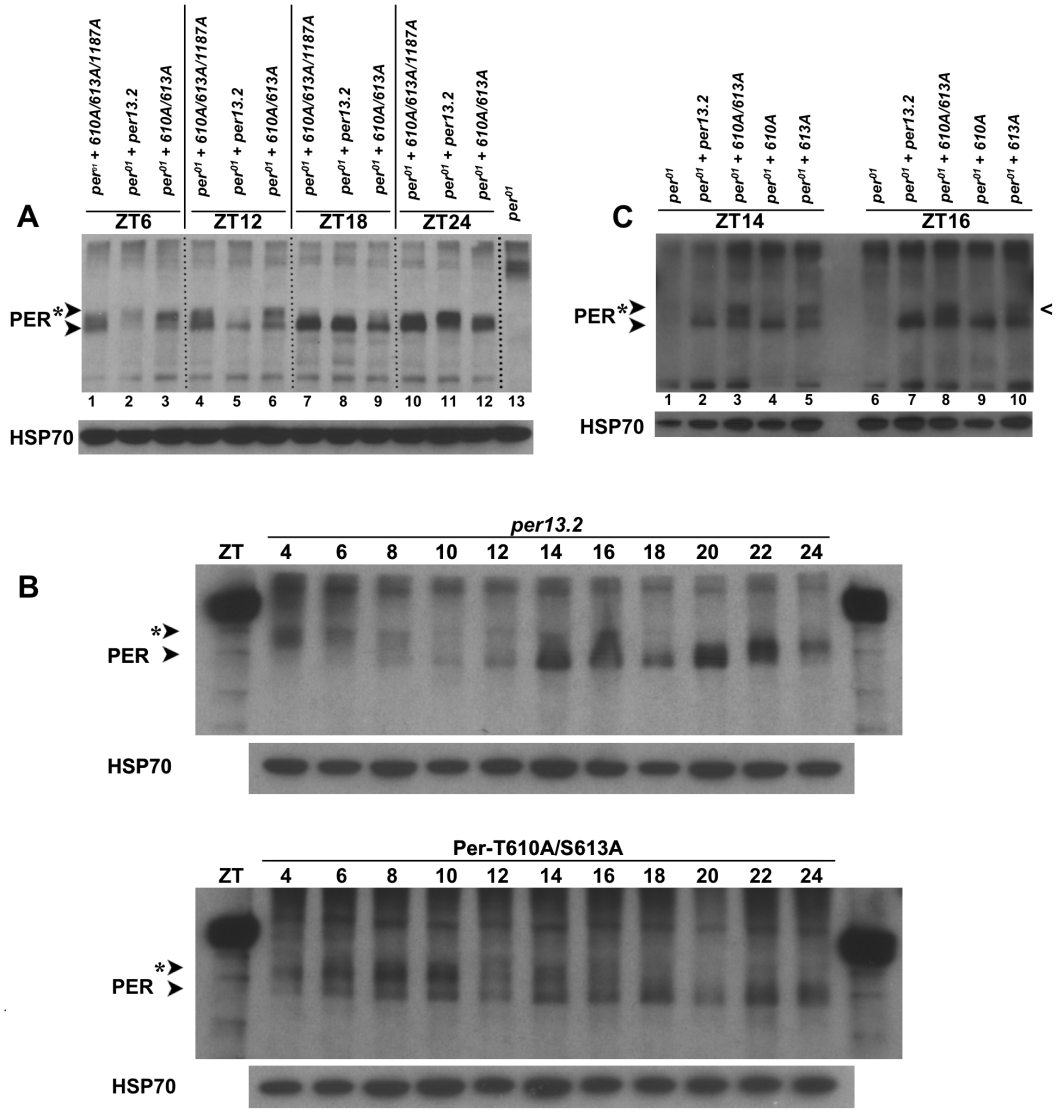
PER-T610A/S613A exhibits defects in protein phosphorylation and stability. (**A**) Head extracts were prepared from an equal number of *per^01^* flies expressing a wild type *per*13.2, PER-T610A/S613/S1187A, or PER-T610A/S613A transgene at the indicated time points. At ZT6, wild type PER (lane 2) is hyperphosphorylated and is declining in levels while both mutant forms of PER are hypo-to-moderately phosphorylated and appear stabilized (lanes 1 and 3). At ZT12, strictly hypophosphorylated wild type PER, presumably representing a new round of synthesis, is observed (lane 5) while mutant PER remains hypo-to-moderately phosphorylated and stable. By ZT18, mutant PER also appears in a hypophosphorylated form, though a small smear in mutant lanes is consistently observed. At ZT24, wild type PER (lane 11) is already becoming moderately phosphorylated while the mutant forms remain hypophosphorylated. As a control, *per^01^* flies without a transgene are shown in lane 13. Similar results were obtained for at least three independent experiments. (**B**) A time course comparison between wild type PER (*per*13.2) and PER-T610A/S613A. Head extracts were prepared from an equal number of flies every two hours at the indicated time points. When levels of wild type PER levels are lowest (top blot, ZT8-12), PER-T610A/S613A levels remain high; lowest level of PER-T610A/S613A expression is most consistently seen between ZT16-20. Note the smear at ZT14 and ZT16 in the *per*13.2 blot is not representative of phosphorylation but rather an artifact in those lanes. (**C**) The single PER-T613A mutant becomes hypophosphorylated earlier in the day than does the PER-T610A/S613A double mutant. Notice at ZT16, the single mutant (last lane) is almost completely hypophosphorylated (caret) while the double mutant remains moderately phosphorylated at this time point. Similar results were obtained from two different experiments.

In an attempt to determine how the T610 and/or S613 mutation(s) affect the well-known cyclic phosphorylation profile of PER, we collected heads at discrete time points throughout the light∶dark cycle from flies carrying a wild type transgene or one containing each single or combined double mutations and performed western blots. Consistent with previously published data, we found that wild type PER is at its lowest levels and hypophosphorylated during the end of the light phase (∼ZT8-12, Figure 5A and B) [18]. Accumulation and phosphorylation increase over the course of the night, with most highly phosphorylated forms found early in the day (ZT4-6; Figure 5A and B). In contrast, PER containing an alanine substitution at T610 and S613 is found in hypo- and moderately-phosphorylated forms early in the day (Figure 5A; compare mutant proteins in lanes 1 and 4 or lanes 3 and 6 to wild type *per*13.2 in lanes 2 and 5) and shows increased phosphorylation over the course of the day (Figure 5B). Mutant PER is hypophosphorylated and at very low levels around ∼ZT18-20, a much later time point than that observed in wild type (Figure 5A; compare mutant protein in lane 7 to *per*13.2 in lane 5), and is at its lowest levels at ZT20 (Figure 5B). Thus, phosphorylation and protein degradation are delayed, presumably leading to a delay in the next cycle, consistent with the long period phenotype observed in flies carrying the double mutant transgene. Interestingly, unlike wild type PER, we noticed that PER-T610A/S613A never becomes completely hyperphosphorylated; we reliably see a hypophosphorylated band at all time points throughout the day (Figure 5A, lanes 1, 4, 7, 10 and 5B). Together, these data suggest that T610 and S613 are critical for normal PER phosphorylation and degradation and are in agreement with the generally accepted notion that phosphorylation is critical for timely PER turnover.

Interrogation of T610A and S613A single mutants revealed that defects observed in the phosphorylation state of the PER-T610A/S613A double mutant are mainly attributable to the mutation at S613 (Figure 5C and data not shown), once again confirming that this residue is a key regulator of PER function. However, we noticed that the single S613A mutant becomes hypophosphorylated ∼2 hours before the double mutant (Figure 5C, compare lanes 3 and 5 at ZT14 as well as lanes 8 and 10 at ZT16; black caret), which is consistent with the longer period observed in the double mutant (∼29–30 hours) as compared to the S613A single mutant (∼26–27 hours). Together, these data support the idea that both residues cooperate to control the overall timing of the circadian cycle with S613 playing a large role.

### PER-T610A/S613A exhibits extended expression in the small LN_v_s

The western analysis above suggests that mutations at T610 and S613 alter the phospho-status of PER and its subsequent degradation, as assayed in whole heads, thereby delaying the clock. However, much of the signal in heads derives from clock protein expression in the eye, which does not directly contribute to behavioral rhythms. Therefore to determine how mutations at T610 and/or S613 affect PER expression and subcellular distribution specifically in central clock cells of the brain, we performed a detailed immunohistochemical analysis (time points every two hours) of PER throughout the day. We first optimized the PER antibody staining protocol and found that varying fixation times can greatly impact the reliability of the PER signal. Thus, for the PER antibody used in this study (UP1140), we incubated fly brains in freshly prepared 4% paraformaldehyde for no more than 15–20 minutes at room temperature. We found that incubation times approaching 45 minutes greatly reduce PER intensity and the results overall become less reliable.

**Figure 6:**
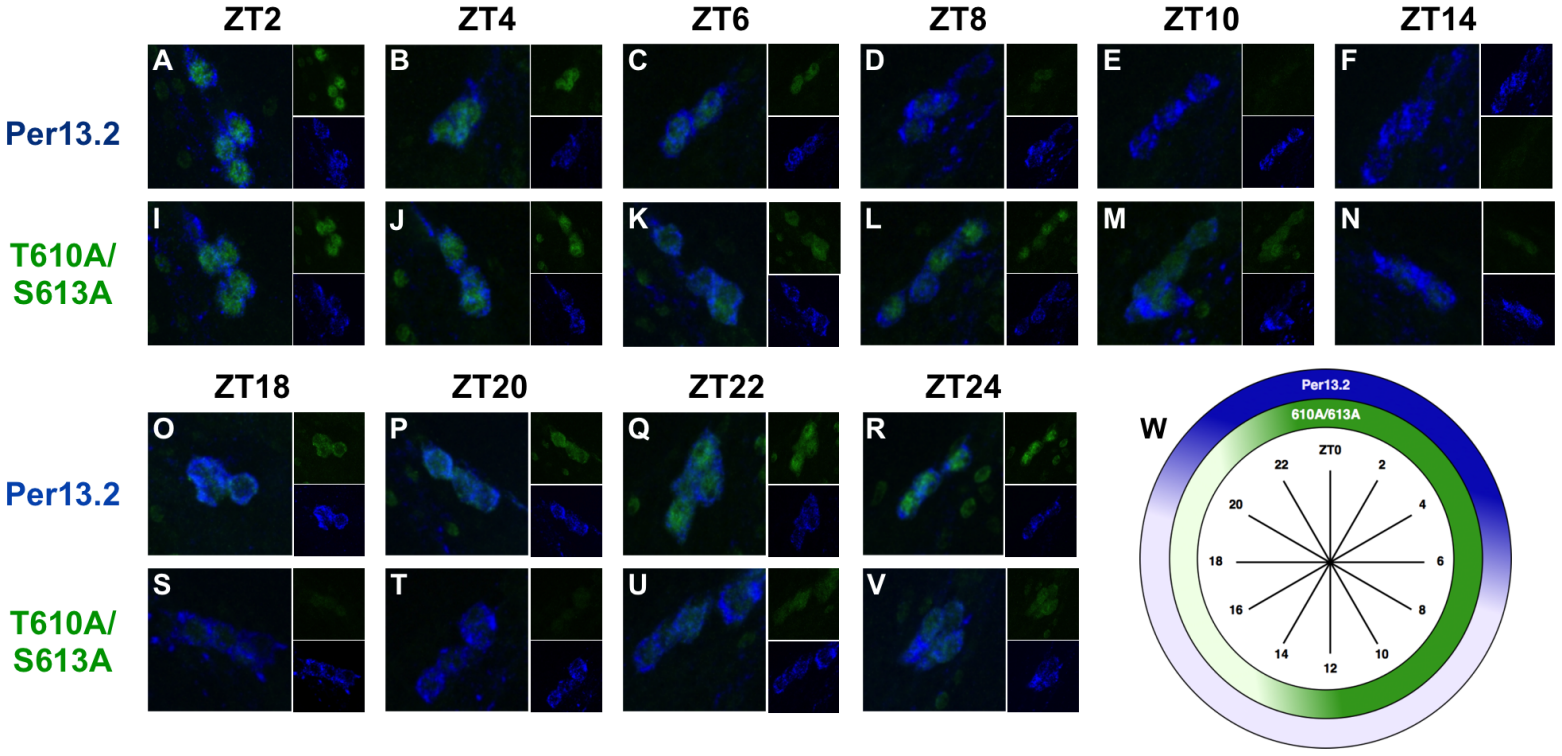
PER-T610A/S613A displays prolonged expression in the small LN_v_s. Brains from male *per^01^* flies expressing the indicated transgene were stained with anti-PER (green; UP1140) and anti-PDF (blue; HH74). Small ventral lateral neurons (LN_v_s) are shown in each panel. ZT time points are indicated at the top of each set of panels. (**A–F, O–R**) Wild type PER (*per*13.2) accumulates in the nucleus of small LN_v_s starting at approximately ZT22, is highly expressed and nuclear by ZT0, and is largely absent by ZT8. (**I–N, S–V**) PER-T610A/S613A exhibits prolonged expression during the day, ZT2-14, and is turned-over at ∼ZT14, much later than wild type PER. Additionally, the double mutant protein appears to accumulate slightly slower than wild type (compare time points at ZT22 and ZT24) (**W**) Diagram depicting the highest levels of PER-13.2 (dark blue) and PER-T610A/S613A (dark green) throughout the day.

The best studied of the central clock cells are the large and small ventral lateral neurons (LN_v_s), which express the neuropeptide, pigment dispersing factor (PDF), while other dorsal neurons also help to modulate circadian behavior in response to light [19], [20]. As the small LN_v_s are the most important cells for driving rhythms in constant darkness, we monitored PER expression in these cells every two hours throughout a 24-hour cycle in both wild type and T610A/S613A mutant flies. As previously reported [21], we found that wild type PER becomes nuclear late at night (∼ZT20-22) and remains predominantly nuclear until ∼ZT6, after which time levels decline (Figure 6A–R, W). Similar to the wild type transgenic protein, PER-T610A/S613A exhibits robust nuclear accumulation in the small LN_v_s from ∼ZT2–ZT6 (Figure 6I–K). However, in contrast to the wild type protein, the double mutant protein is not degraded between ZT6-8 but instead remains in the cell until ∼ZT14, after which time it is degraded. Additionally, we noticed that the double mutant protein accumulates more slowly than wild type (Figure 6, compare panels at ZT22 and ZT24). This is most likely due to the prolonged inhibition of transcription and thus a delay in re-initiation of the next molecular cycle. We conclude that the major defect in the mutant is increased stability and extended expression of PER during the day, which leads to delayed accumulation of *per* RNA and protein in the next cycle.

### CLK phosphorylation and CLK-mediated output are defective in PER-T610A/S613A mutants

**Figure 7:**
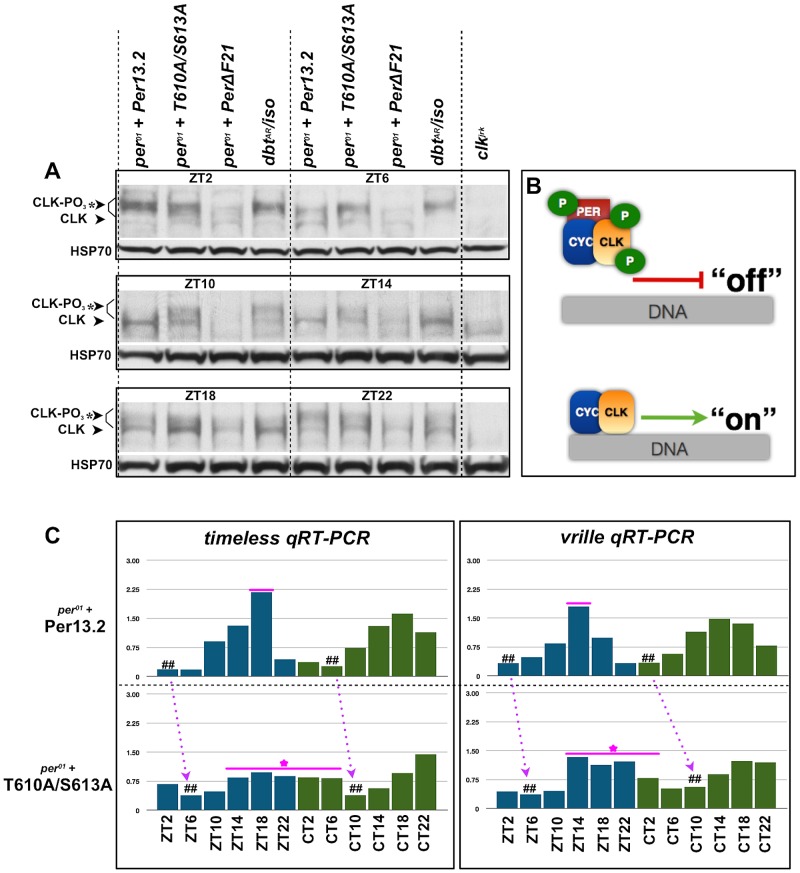
The T610 and S613 mutations affect CLK phosphorylation and CLK-mediated transcriptional output. (**A**) Head extracts were prepared from an equal number of *per^01^* flies expressing a wild type *per*13.2 or PER-T610A/S613 transgene at the indicated time points. As a control to demonstrate CLK hypophosphorylation and hyperphosphorylation at each time point, we also prepared head extracts from *per^01^* flies expressing a PER-ΔDBT (lacks DBT binding; line F21, a gift from Paul Hardin; [22]) or a catalytically compromised mutant form of DBT, *dbt^AR^/+*, respectively. At ZT2, CLK is exclusively hyperphosphorylated in both wild type and PER-T610A/S613A, consistent with low transcriptional output. By ZT6, CLK is partially de-phosphorylated in files expressing wild type PER, whereas CLK remains exclusively hyperphosphorylated in the double mutant. From ZT10–ZT14, CLK is exclusively hypophosphorylated in flies expressing the wild type transgene and becomes mostly hyperphosphorylated once again by ZT22. On the other hand, in double mutant flies, CLK is observed as a mixture of hypo- and hyperphosphorylated forms from ZT10–ZT22. (**B**) Diagram depicting the effects of CLK phosphorylation on transcriptional output. (**C**) Quantitative real-time RT-PCR was used to measure *tim* and *vri* mRNA levels in heads from flies expressing wild type *per*13.2 or PER-T610A/S613A at the indicated ZT and CT time points. Dashed arrows designate a shift in daily troughs of CLK-mediated output (number signs) between wild type and mutant flies, consistent with the long period phenotype. Magenta line+star represent delayed transcriptional repression and blunted transcriptional response in the double mutant. Data are representative of multiple independent experiments.

Previous reports demonstrate that CLK phosphorylation is coincident with nuclear PER accumulation. Additionally, it's been documented that CLK-mediated transcriptional output is repressed when CLK is hyperphosphorylated and high when CLK is hypophosphorylated (Figure 7B) [22]. Therefore, we sought to investigate the status of CLK phosphorylation and CLK-mediated transcriptional output in PER-T610A/S613 double mutant flies. To determine the phospho-status of CLK in double mutant flies, we collected heads from flies carrying a wild type or double mutant transgene at discrete time points throughout the light∶dark cycle and performed western blots with an anti-CLK (GP50) antibody ([23]; a gift from Paul Hardin). As predicted, flies expressing the wild type transgene exhibit hyperphosphorylated CLK late at night and into the early morning (ZT22 and ZT2) [23], which is consistent with low levels of CLK-mediated transcription (Figure 7A and C). Hypophosphorylated CLK is observed from ZT6 onwards (Figure 7A), and this is associated with a new round of CLK-mediated transcription between ZT6-18 (Figure 7C). In contrast, in flies expressing PER-T610A/S613A, CLK remains completely hyperphosphorylated until at least ZT6 (Figure 7A), consistent with the observed delay in CLK-mediated output (Figure 7C, number signs and magenta arrows). Additionally, in mutant flies, CLK is detected at many time points, in particular from ZT10 to ZT22, as a mixture of hyper- and hypophosphorylated forms (note the two bands in the double mutant lanes in Figure 7A). The presence of both forms at these times is expected to blunt the peak expression of CLK targets, which is indeed the case for *timeless* and *vrille* (Figure 7C). CLK is only observed exclusively in its hyperphosphorylated state at ∼ZT2-6, concomitant with the trough in CLK-mediated transcription (Figure 7C).

### Mutations in the Per-Short domain are epistatic to S613A

**Figure 8:**
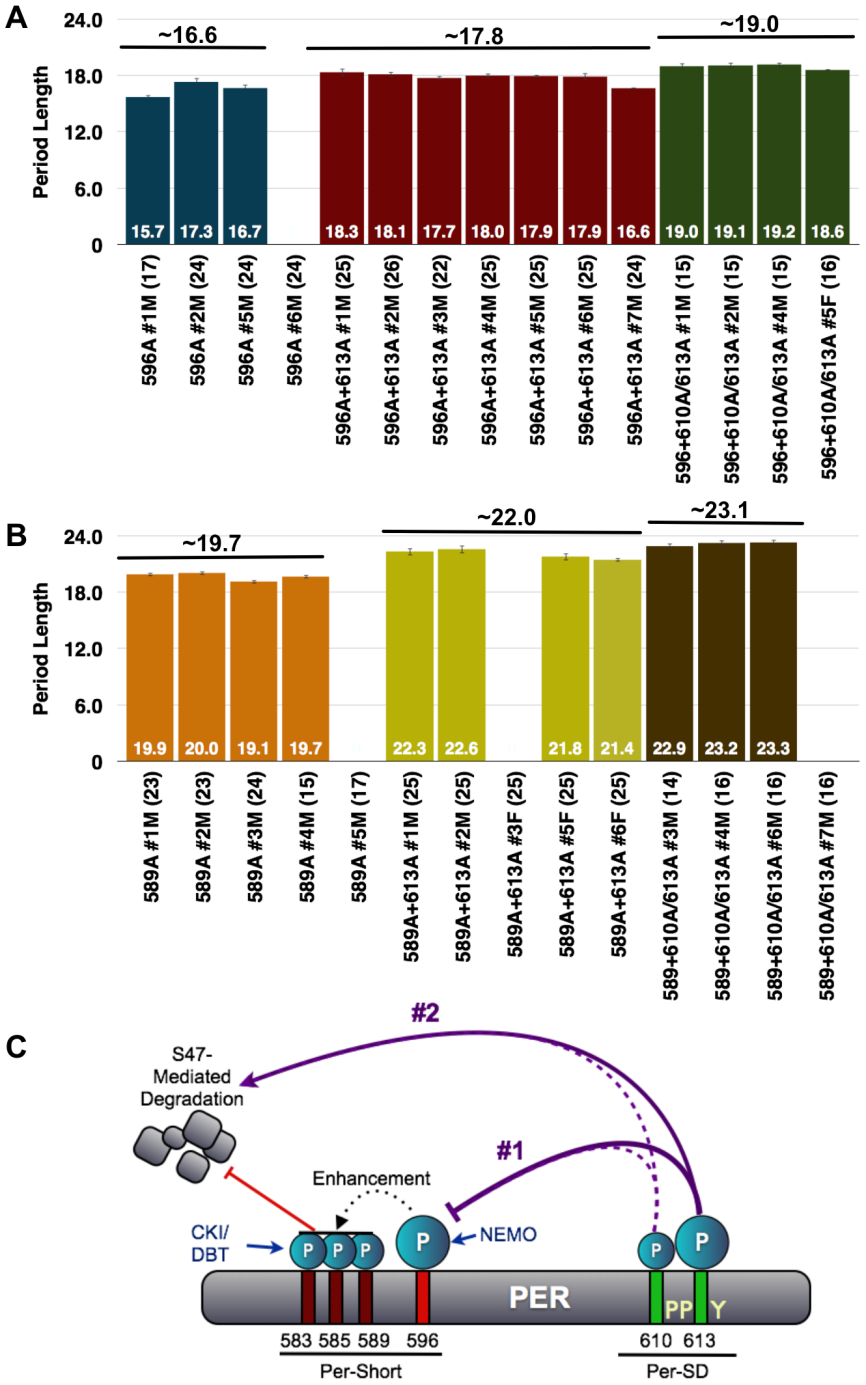
The short period phenotype of S596A is epistatic to S613A. (**A**) Graphs depicting period length of *per^01^* flies carrying transgenes harboring the single S596A mutation (blue bars) or S596A combined with the S613A (red bars) or T610A/S613A (green bars) mutations. Period lengths are listed within each bar and values in parentheses indicate the number of flies analyzed. Average period length for all lines combined is listed above each set. Raw data are summarized in Table 1. Only male flies were analyzed in these experiments. Note, the S596A+S613A lines possess period lengths similar to those of S596A single mutant lines suggesting that S596A is largely epistatic to S613A; in other words, without S596, regulation by S613 is not critical. Additionally, S596A is also largely epistatic to the long period phenotype of the double T610A/S613A mutant. (**B**) The S613A (yellow bars) and T610A/S613A (brown bars) mutations are able to more dramatically increase the period length of the S589A mutation (orange bars), which is only one of three residues regulated by 596. These data are consistent with S613A regulating the more critical phospho-residue, S596. (**C**) Updated model representing potential regulation of PER by the PER-Short and PER-SD domains. In scenario #1, the PER-SD domain regulates phosphorylation of residues within the PER-short domain, while scenario #2 proposes that phosphorylation of the PER-Short and PER-SD domains control clock speed in parallel.

A mutation at serine-589 of PER, or in amino acids located close to S589, produces a short-period phenotype, leading to the designation of this region as the Per-short domain [24]. Recently, it was reported that the Per-short domain extends to S596, and phosphorylation within this region regulates PER turnover by inhibiting phosphorylation at S47, a site recognized by the E3 ligase, SLIMB [25]. Phosphorylation at S596 by the NEMO kinase is especially critical as it enhances phosphorylation at upstream residues, S583, S585, and S589, thereby delaying PER degradation. As S613 is located close to the Per-Short domain and mutations in this residue yield the stronger of the two single mutant phenotypes, we generated S596A-S613A double mutant flies. As shown in Figure 8A, the PER-S596A-S613A transgene results in a period length only slightly longer than that produced by the S596A transgene alone, indicating an interaction between these two phospho-sites as independent mechanisms would result in additive effects on period. Instead, the short-period phenotype of the S596A mutation is largely epistatic to the long period of S613A, suggesting that phosphorylation at S596 is downstream of phosphorylation at S613.

Given that T610A-S613A mutant flies have a more severe phenotype than do single S613A mutants, we also generated flies with transgenes containing mutations at T610/S613 in combination with S596A and compared them to flies containing transgenes harboring single mutations. The stronger T610A/S613A double mutant combination with S596A also shows a dominance of the short period phenotype associated with S596A. Thus, while T610A/S613A together increase the period of the S596A single mutant by approximately 2.5 hours (Figure 8A), this is still much less than what would be predicted for an additive interaction. These data support the idea that the mutation at S596 is epistatic to and downstream of the T613/S610 double mutation. Interestingly, S613A alone and T610A/S613A are able to more significantly increase the period length of the single S589A mutation (Figure 8B), perhaps because phosphorylation at S596 can still enhance phosphorylation at S583 and S585. We propose that regulation of S596 by S613 is critical and that phosphorylation at T610 also plays a minor role. However, our data also suggest that S613, and perhaps more likely T610, play an independent role in regulating period length given the modest increase observed when the S596A mutation is combined with both of these mutant residues. As the S613A mutation increases period length, while S596A decreases period, we propose that phosphorylation at S613 normally inhibits phosphorylation at S596, though alternative models also exist (Figure 8C).

## Discussion

Our data provide functional evidence for a region of PER suggested previously by studies in S2 cells, namely the Per-Short Downstream (Per-SD; [17]) domain. We show that this domain is regulated by phosphorylation and interacts with other phosphorylated regions to control circadian period. Using behavioral, biochemical and immunohistochemical analyses, we demonstrate that within this domain, T610 and S613 act cooperatively to generate a functional circadian clock in *Drosophila* although S613 plays a larger role in determining circadian period. Our experiments and observations also indicate that aberrant phosphorylation and slower rates of degradation are responsible for generating the long period associated with the T610A/S613A mutations. And while phospho-mediated regulation of PER is well established, our data suggest a new twist on this idea. We propose that both phospho-residues, T610 and S613, cooperate to regulate PER turnover, such that one can substitute for the other to a large extent, but mutation of both dramatically lengthens circadian period. We were able to uncover this cooperative interaction due to the fact that we initially mutated groups of amino acids within PER. Simply looking for effects based on mutating individual residues would not have revealed this interaction.

While some of the phospho-residues identified in this study overlap with those recently published [11], [14], [16], [17], others do not. Reasons for these minor discrepancies are quite apparent. For example, in this study, we treated S2 cells with phosphatase inhibitors thereby attempting to retain most phospho-marks within PER regardless of the kinase involved. On the other hand, previous studies were specifically focused on identifying DBT- or SGG-directed residues. Additionally, the Kivimae et al. (2008) study used *in vitro* generated DBT and a GST-PER fusion to identify phospho-marks. Given these differences in experimental approach it's not surprising that some of our sites overlap while others do not. Ultimately determining a complete PER phospho-map will be extremely useful for the circadian field. Table 2 provides an updated summary of currently identified phospho-residues in *Drosophila* PER.

**Table 2:** Summary of identified phospho-residues on PER (including this study).

Amino Acid	Predicted Kinase	Reference	Mutant Behavioral Phenotype
S42	CKI/DBT	Chiu, et al., 2008; This study	
S47	CKI/DBT	Chiu, et al., 2008	Long Period
S59	CKI/DBT	Chiu, et al., 2008	
S60	CKI/DBT	Chiu, et al., 2008	
S93	GSK/SGG	Ko et al., 2010; This study	
S97	GSK/SGG	Chiu, et al., 2008; Ko et al., 2010; This study	
S109		This study	
S132	CKI/DBT	Chiu, et al., 2008	
S149	CK2	Lin et al., 2005; Chiu, et al., 2008; This study	Slightly Longer Period
S151	CK2	Lin et al., 2005; Chiu, et al., 2008; This study	Slightly Longer Period
S153	CK2	Lin et al., 2005; Chiu, et al., 2008; This study	Slightly Longer Period
S164	GSK/SGG	Ko et al., 2010	
S169	GSK/SGG	Ko et al., 2010	
S174	GSK/SGG	Ko et al., 2010; This study	
S177		This study	
S199		This study	
T207		This study	
T219		This study	
T228		This study	
S516		This study	
S542		This study	
T583	CKI/DBT	Ko et al., 2010; Chiu, et al., 2011; This study	
S585	CKI/DBT	Chiu, et al., 2008; Ko et al., 2010; Chiu, et al., 2011; This study	Short Period
S589	CKI/DBT	Kivimae et al., 2008; Chiu, et al., 2008	Short Period
S596	NEMO	Chiu, et al., 2008; Ko et al., 2010; Chiu, et al., 2011; This study	Short Period
S607		Kivimae et al., 2008	
T608		This study	
T610		Chiu, et al., 2008; Kivimae et al., 2008; Ko et al., 2010; This study	Long Period
S613		Kivimae et al., 2008; This study	Long Period
S629		Kivimae et al., 2008	
T633		This study	
S657	GSK/SGG	Ko et al., 2010	Slightly Longer Period
S661		Ko et al., 2010	Slightly Longer Period
S773		Chiu, et al., 2008; This study	
T808		This study	
S815		This study	
S826	GSK/SGG	Chiu, et al., 2008; Ko et al., 2010; This study	
S828	CKI/DBT	Chiu, et al., 2008; Ko et al., 2010	
S865		This study	
S876		Chiu, et al., 2008; This study	
T883		Chiu, et al., 2008; Ko et al., 2010; This study	
T889	CKI/DBT	Chiu, et al., 2008	
T978	CKI/DBT	Chiu, et al., 2008	
S981		Chiu, et al., 2008; Ko et al., 2010	
T1077		This study	
S1080		This study	
S1102		This study	
S1103		Chiu, et al., 2008; This study	
S1130	CKI/DBT; GSKSGG	Chiu, et al., 2008; Ko et al., 2010	
S1133		Kivimae et al., 2008	
S1148		This study	
S1185	GSK/SGG	Ko et al., 2010	
S1187	GSK/SGG	Ko et al., 2010; This study	
S1204,5,6	GSK/SGG	Ko et al., 2010	
T1219		Kivimae et al., 2008	

### The Per-Short Downstream (Per-SD) domain

T610 and S613 were previously identified as phospho-acceptor sites [14], [16], [17], and Kivimae at al (2008) designated this region the Per-Short Downsteam (Per-SD) domain. We retain this nomenclature and show that this region is critical for proper PER function. In addition to the original sites identified in this study (T610 and S613), we also demonstrate that proline-611 and proline-612 as well as tyrosine-614 are critically important for proper time-keeping mechanisms. As suggested by Kivimae et al. (2008), the arrangement of phospho-residues at 604, 607, 610, and 613 resembles a CKI phosphorylation motif found in human hPer2 that has been implicated in hFASPS, consistent with the idea that the T610/S613 cluster is relevant for rhythms across species. Accordingly, we note that S613 and Y614 are highly conserved from flies to humans.

### Biochemical and cellular basis of the T610A/S613 mutant phenotype

On a molecular and biochemical level, doubly mutated PER exhibits increased stability, manifest as prolonged expression of hyperphosphorylated PER. Note, prolonged hyperphosphorylation of PER is consistent with a mechanism by which phosphorylation within the Per-SD domain reduces phosphorylation at additional residues, for example, those within the Per-Short domain, in particular S596. In the absence of phosphorylation at S613, PER remains partially hyperphosphorylated, likely due to increased phosphorylation at S596 and other residues of the Per-short domain. Double mutant flies also display extended CLK hyperphosphorylation, which is expected based upon the reported effects of PER on CLK phosphorylation during the repression phase of the cycle [26]. This ultimately delays the trough of CLK-mediated transcriptional output and thus the next molecular circadian cycle.

Our immunohistochemical analyses of T610A/S613A double mutant flies demonstrate that in the small LN_v_s, PER protein expression is extended later in the day leading to a slight delay in protein accumulation of the next molecular cycle. As small LN_v_s are the primary determinants of free-running rhythms, we attribute the behavioral defects to these deficiencies in the LN_v_s. Since extended nuclear PER expression is usually correlated with CLK-mediated transcriptional repression our data suggest that CLK-mediated transcription should remain low for an extended period throughout the day. However, we observe prolonged expression of CLK targets, probably because the daily decline in CLK-mediated transcription is also delayed. Interestingly, the same phenotype is seen in *tim^UL^* mutants, which have a similar long period [27]. The prolonged expression and lower peak levels of CLK targets in the double mutants, relative to those in flies expressing a wild type PER transgene, may be due to the presence of hypo- and hyperphosphorylated CLK (these have opposing effects on transcription) at times of transcriptional activity.

### How do T610 and S613 set the pace of the *Drosophila* clock?

It is well documented that PER phosphorylation plays a pivotal role in setting the pace of the circadian clock, and more recently, progress has been made in teasing apart the intricacies of this regulation [28]. For example, previous data demonstrated that phosphorylation at S47 is critical for binding of the E3 ubiquitin ligase, SLIMB, and subsequent proteasomal degradation [16], [25]. Furthermore, phosphorylation at S661 by an unknown kinase enables subsequent phosphorylation of S657 and promotes entry of PER into the nucleus but not degradation [14]. Likewise, phosphorylation by CK2 regulates timing of nuclear entry [8], [9]. In addition, mutating residues within the Per-Short phospho-cluster (for example, the original *per^short^* allele) leads to a decrease in daily period length [24]. As noted previously, Chiu et al. (2011) demonstrated that NEMO and DBT phosphorylate residues within the Per-Short domain, including S585, S589, and S596, thereby blocking phosphorylation at S47 and setting the molecular clock to the correct speed by delaying PER degradation. Thus, it is clear that phosphorylation can affect PER in many different ways (rate of turnover, repressor function, localization, etc) and not all phospho-marks ultimately lead to PER decline.

Therefore, how do the phospho-residues identified in this study, T610 and S613, impose regulation? Given the close proximity of T610 and S613 to the Per-Short domain, it was not unreasonable for us to imagine some sort of interaction between these two clusters of phospho-residues. Indeed, the epistasis data presented in Figure 8 suggest a model in which phosphorylation at S613 (and perhaps T610) could regulate phosphorylation at S596, which in turn affects phosphorylation at S585 and S589, and ultimately S47. However, we acknowledge that our data are also consistent with 610/613 controlling the speed of the clock in parallel, yet in opposition to, the regulation imposed by phosphorylation of the Per-Short domain. For example, it is possible that mutations at S589 and S596 reduce the time interval during which mutations at 610 and/or 613 act, thereby reducing their effects on period length. In Figure 8, we highlight both of these scenarios; each is consistent with the increased stability of PER and the long-period phenotype observed in the S613A mutant flies.

From the data presented in Figure 3, it is clear that T610 cooperates with S613 to control the speed of the clock. Interestingly, this interaction may, to some extent, be independent of the proposed role for S613 in regulating phosphorylation at S596. We base this on the finding that the 610 mutation, despite producing essentially no phenotype by itself, significantly increases not only the period of the 613A mutant, but also of the 613A-596A double mutant. It is possible that 610 alters the repressor activity of PER, as was suggested by a previous report [17]. In this report, the authors proposed that the Per-Short domain regulates the Per-SD domain, which is the reverse of what we propose here. However, we note that the previous study did not examine 613 or 596, and also focused specifically on the repressor activity of PER in S2 cells and not on protein stability in flies, which is the major phenotype reported here. As described above, we find that effects on PER repressor function in S2 cells do not always translate into period effects *in vivo*. Overall, we believe our genetic epistasis experiments support our current view. Regardless, it is clear that the Per-SD domain is an important regulatory region, and appears to function upstream of or in parallel to previously identified functional domains of PER in controlling circadian period. Our findings suggest that a series of interactions between different domains of PER regulate its phosphorylation and its ultimate degradation to yield precise cycles of protein expression and of behavior.

## Materials and Methods

### Mass spectrometry and identification of phospho-residues

#### Induction of PER phosphorylation in S2R+ cells and purification of PER protein by immunoprecipitation (IP)

p*Act*-*per*-HA transfected S2R+ cells were treated with vehicle (DMSO), 2 µM Tautomycin (Calbiochem), or 50 nM Okadaic Acid (Biomol), along with MG132 (100 µM, Sigma) for 4 hrs before harvest. Cells were then lysed and immunoprecipitated with anti-HA (Covance) antibody as described previously [13]. Immune complexes were eluted in 4xLDS Sample Buffer (Invitrogen) and run on a 3–8% tris-acetate gel (Invitrogen). PER protein bands were visualized by Coomassie-blue staining (Figure 1).

#### Sample preparation, acquisition, and mass spectrometry analysis PER

Protein samples were excised from the Coomassie-blue gel and subjected to in-gel digestion with sequencing grade 12.5 ng/µl trypsin (Promega) in 50 mM ammonium bicarbonate pH 8.2 overnight at 37°C. Peptides were extracted with 50% acetonitrile (MeCN), 5% formic acid (FA) and then dried. Dried peptides were resuspended in 10 µL of 5%MeCN, 4% formic acid (FA), and 2 µL was loaded onto a pulled fused silica microcapillary column packed with C18 reverse-phase resin (Magic C18AQ; 5-µm particles; 200-Å pore size; Michrom Bioresources) using a Famos autosampler (LC Packings). Once loaded, the peptides were separated using an Agilent 1100 series binary pump across a 45 min linear gradient of 6–28% CH3CN in 0.125% HCOOH at a flow rate of 600 nL/min.

Mass spectra were acquired in an LTQ-Orbitrap hybrid mass spectrometer (Thermo Fisher) over the entire run using ten MS/MS scans following each survey scan. Raw data were searched against the HA-Per amino acid sequence using Sequest software with no enzyme specificity and a 1.1 Da mass tolerance. The search parameters used for post-translational modification included a static modification of 57.02146 Da on cysteine (carboxyamidomethylation) and dynamic modifications of 15.99491 Da on methionine (oxidation) and 79.96633 Da on serine, threonine, and tyrosine (phosphorylation). The probability of correct phosphorylation site localization was determined for every site in each peptide using the AScore algorithm as described in [29].

### Cell culture experiments

#### Expression constructs and site-directed mutagenesis

Site-directed mutagenesis of PER phosphorylation sites was conducted using QuikChange (II, Multisite, or Lightening) Site-Directed Mutagenesis Kits (Stratagene/Agilent). Primers were designed using Agilent's online primer design tool. p*Act-per*-HA (a gift from S.M. Reppert) was used as a template for mutagenesis.

#### *per-luciferase* assay

For the luciferase assay [30], S2R^+^ cells were cultured as described above but in 96-well plates. 48∼72 hr post-transfection, cells were lysed and processed using the Dual-Glo Luciferase Assay System (Promega) according to the manufacture's protocol using the Victor3V luminometer (PerkinElmer). Firefly luciferase (*per-luc*) activity was normalized to Renilla luciferase activity. For transfection of the cells in each of the 96-wells, 10 ng p*Act*-r-*luc*, 20 ng *per*-E-*luc*, 10 ng p*Act*-*Clk*-HA, and 5 ng WT and mutant p*Act-per*-HA were used.

#### Phosphatase inhibition in S2R^+^ cells and Western blots

S2R^+^ cells were transfected with expression constructs using Cellfectin (Invitrogen) as described in the product manual. For the phosphatase inhibition assay, cells were co-transfected with indicated amount of p*Act*-*Nipp1*-V5 [13], or incubated in medium containing indicated amounts of PP2A inhibitor, Okadaic Acid (OA; Biomol) or vehicle DMSO for 4 hours before harvest. 48 hr post-transfection, cells were harvested and processed for western blot analysis. Primary antibodies were used at the following dilutions: anti-HA (Covance) 1∶1000, anti-V5 (Invitrogen) 1∶1000, and anti-HSP70 (Sigma) 1∶20,000.

### Fly experiments

#### Fly stocks

The following transgenic lines were generated by BestGene, Inc (2918 Rustic Bridge,Chino Hills, CA 91709; See below): *per13.2, per-T610A/S613A/S1187A, per-T610A/S613A, per-T610A, per-S613A, per-S1187A, per-P611A, per-P612A, per-Y614A, per-S596A, per-S589A, per-S596A/S613A, per-S589A/S613A, per-S596A/T610A/S613A, per-S589A/T610A/S613A*. Males of these transgenic lines were crossed to *yw*, *per^01^* females and circadian rhythms from male hemizygous progeny were analyzed in all experiments.

#### Site directed mutagenesis, transgenic flies, and rescue assay

Site-directed mutagenesis of genomic per phosphorylation sites was conducted using QuikChange (II, Multisite, or Lightening) Site-Directed Mutagenesis Kits (Stratagene/Agilent). Primers were designed using Agilent's online primer design tool (Table S1). A *Bgl*-II-*per* genomic fragment contained within the pSP73 vector was used as a template for mutagenesis. Following mutagenesis and sequence confirmation, the *Bgl*-II-*per* fragment was subcloned into the pCasPeR-*per*-13.2 (HA/His tagged) vector [31]. Wild Type or mutant transgenic lines were generated in the *w^1118^* background by BestGene, Inc (2918 Rustic Bridge, Chino Hills, CA 91709). Individual lines were tested for rescue of the *per^01^* mutant phenotype using the traditional TriKinectics *Drosophila* Activity Monitor (DAM) setup. Data from each individual transgenic line are shown in the paper. For all rescue experiments, a fly was considered rhythmic if the FFT value was greater than 0.02.

#### Western blot analysis from fly heads

2–3 day old male flies (*per^01^/Y +/−* transgene) were entrained to the appropriate LD/DL light schedule for a minimum of 3 full cycles prior to collection on day 6. At the indicated time points, fly heads were collected on dry ice and immediately homogenized on wet ice in 1× Lysis Buffer [1× Passive Lysis Buffer (Promega), 1× Complete EDTA-free Protease Inhibitor (Roche), 20 nM Okadaic Acid, 5 mM NaF, 25 nM DTT (Pierce)]. For PER westerns, lysates were spun down, boiled, run on 6% Tris-Glycine gels (Novex; Life Technologies), transferred to nitrocellulose membranes and probed with the following antibodies: anti-PER (UPR1139; 1∶2000) or anti-HSP70 as loading controls. For CLK westerns, lysates were spun down, boiled, run on 3–8% Tris-Acetate gels (Novex; Life Technologies), transferred to nitrocellulose membranes and probed with the following antibodies: anti-CLK (GP50; 1∶4000, a gift from Paul Hardin) or anti-HSP70 as loading controls.

#### Quantitative RT-PCR

Fly heads were collected on dry ice at indicated time points in LD or DD. Total RNA was isolated using Trizol isolation system (Life Technologies), and cDNAs were synthesized by using a high-capacity cDNA Reverse Transcription kit (Applied Biosystems). Quantitative real-time PCR was performed in an ABI prism 7100 using a SYBR Green kit (Applied Biosystems) with gene specific primers. The following primer pairs were designed according to [22]: *rp49*-5′-TACAGGCCCAAGATCGTGAA-3′ and 5′-GCACTCTGTTGTCGATACCC-3′; for *vri* were 5-ATGAACAACGTCCGGCTATC-3′ and 5′-CTGCGGACTTATGGATCCTC-3′; and for *tim* were 5′-GGTG GCATCTGTGTACGAAA-3′ and 5′-GATCTCGGTTCGCTCAAGTC-3′. For each sample, RNA quantity was determined by the standard curve for each gene and normalized to *rp49*.

#### Immunohistochemistry

2–3 day old male flies (*per^01^/Y +/−* transgene) were entrained to the appropriate LD/DL light schedule for a minimum of 3 full cycles prior to dissections on day 6. At the indicated time points, fly heads of each genotype were gently opened and immediately exposed to 4% paraformaldehyde (in 1× PBS) for 15–20 minutes, followed by incubation in 1× PBST at room temperature for the duration of all dissections at each time point. Note, at each time point, there was no more than a 5–10 minute time delay between when the heads from the first and last genotypes initiated fixation. Dissected brains from each time point remained in 1× PBST at 4 degrees until all time points throughout the day were complete. At the completion of all dissections, brains were incubated with 1× PBST for a ∼30 minutes at room temperature, blocked with Dk-Block (5% Donkey Serum in 1× PBST) for 20 minutes, and incubated with primary antibody overnight in Dk-Block at 4 degrees. The following primary antibodies were used: Guinea Pig anti-PER (UP1140; 1∶1000) and Rabbit anti-PDF (HH74; 1∶500). The next day, following a 30-minute wash in 1× PBST at room temperature, brains were incubated with secondary antibodies for two hours in Dk-Block at room temperature. The following secondary antibodies were used at 1∶500: FITC Dk-anti-Guinea-Pig (1∶500) and Alexa555 Dk-anti-Rb. Samples were then washed for 30–60 minutes in 1× PBST at room temperature, cleared in 70% glycerol, mounted in VectaShield, and stored on the slides at 4 degrees.

#### Confocal microscopy and image analysis

Image stacks containing small LN_v_s from each sample were obtained using a Leica SP5 confocal microscope using a 40× oil-immersion objective and a 0.5 µM step size. Confocal settings for the PDF signal were kept roughly the same for all images, but were adjusted slightly in some cases to obtain a high quality image for analysis; however, at no time was the PER signal changed. Additionally, at no time were the overall signals saturated as determined by QLUT parameters. PDF staining was used to determine the nuclear/cytoplasmic boundaries [21] and a 2D image was generated for analysis by merging Z-planes through each set of cells.

## Supporting Information

Table S1 Primers used in this study to generate mutant PER transgenes. The following primers and mutagenesis kits were used to mutate the indicated regions/residues within the Per13.2 vector backbone. See materials and methods for additional details.
(DOC)
