# Supplementary material for: Cooperative Interaction between Phosphorylation Sites on PERIOD Maintains Circadian Period in Drosophila
Source: PLoS Genet. 2013 Sep 26;9(9):e1003749. doi: 10.1371/journal.pgen.1003749 (PMC3784489; doi:10.1371/journal.pgen.1003749)
Supplement: Table S1 — Primers used in this study to generate mutant PER transgenes. The following primers and mutagenesis kits were used to mutate the indicated regions/residues within the Per13.2 vector backbone. See materials and methods for additional details. (DOC) [file pgen.1003749.s001.doc]

| Region/Site Mutated | QuikChange Kit Used | Mutated Residue | Primer Sequence (lowercase letter = bp change) |
| --- | --- | --- | --- |
| PP2A-1 | MultiSite | 174/177 | AGCTTTCCGATTCCCgCGCCGCTAgCCGTCACCATTGTCCC |
|  |  | 199 | CGCAGCCGGTCTGGACgcCGGGCTGGCCAAGTTC |
|  |  | 207 | CCAAGTTCGACAAGgCCTGGGAGGCAGGTCC |
|  |  | 219 | CAAGCTGGAGTCCATGgCCGGGGTGGGCGCAGC |
|  |  | 228 | AGCGGCGGCGGGCgCAGGGCAGCGCGGGGAG |
| PP2A-2 | MultiSite | 1077/1080 | GGTGTACACGACCgCGCCGGCGgCCATGACGAAGAAGGTG |
|  |  | 1102/1103 | CCCAGGTGCAGCGTCCCgCCgCGCAGAGCGCATCCGTC |
| NLS-2 | MultiSite | 808/815 | TCCGCCAATGACgCCCTTAAGATGCTGGAGTACgcCGGTCCAGGCCACG |
|  |  | 826 | GATAAAAAGAGGAGGCgCCCATTCCTGGGAGGGAG |
| NON-1 | MultiSite | 610/613 | AGAGTTCCACCGAGgCGCCGCCCgcCTACAACCAGCTAAAC |
|  |  | 1187 | ggacggatcggaggctccgccggacacc |
| 610A | Lightning | 610 | gttccaccgaggcgccgcccagc (Sense) |
|  |  |  | gctgggcggcgcctcggtggaac (Antisense) |
| 613A | Lightning | 613 | ccgagacgccgcccgcctacaaccagctaa (Sense) |
|  |  |  | ttagctggttgtaggcgggcggcgtctcgg (antisense) |
| 596A | Lightning | 596 | tgatgctcggcgagattgcgccgcacc (Sense) |
|  |  |  | ggtgcggcgcaatctcgccgagcatca (AntiSense) |
| S589A | Lightning | 589 | ctcggagcgggacgccgtgatgctcggc (sense) |
|  |  |  | gccgagcatcacggcgtcccgctccgag (antisense) |
| P611A | Lightning | 611 | CCACCGAGACgCGCCCAGCTAC (SENSE) |
|  |  |  | GTAGCTGGGCGCcGTCTCGGTGG (ANTISENSE) |
| P612A | Lightning | 612 | ACCGAGACGCCGgCCAGCTACAACC (SENSE) |
|  |  |  | GGTTGTAGCTGGcCGGCGTCTCGGT (ANTISENSE) |
| Y614A | Lightning | 614 | CGAGACGCCGCCCAGCgcCAACCAGC (SENSE) |
|  |  |  | GCTGGTTGgcGCTGGGCGGCGTCTCG (ANTISENSE) |
